# Comparison of the performance of a Three-Dimensional Body Scanner and radiography in evaluating adult scoliosis

**DOI:** 10.7717/peerj.20752

**Published:** 2026-02-03

**Authors:** Ting-Ju Kuo, Chin-Yin Yu, Jui-Chi Lin, Chien-Min Lin, Tsan-Hon Liou, Chih-Wei Peng, Hung-Chou Chen

**Affiliations:** 1 Department of Physical Medicine and Rehabilitation, Shuang Ho Hospital, New Taipei City, Taiwan; 2 Taipei Neuroscience Institute, Taipei Medical University, Taipei, Taiwan; 3 Department of Neurological Surgery, Shuang Ho Hospital, New Taipei City, Taiwan; 4 Department of Surgery, School of Medicine, College of Medicine, Taipei Medical University, Taipei, Taiwan; 5 Department of Physical Medicine and Rehabilitation, School of Medicine, College of Medicine, Taipei Medical University, Taipei, Taiwan; 6 Department of Physical Medicine and Rehabilitation, Wan Fang Hospital, Taipei Medical University, Taipei, Taiwan; 7 Biomedical Engineering, Taipei Medical University, Taipei, Taiwan; 8 School of Gerontology Health Management, College of Nursing, Taipei Medical University, Taipei, Taiwan; 9 Center for Evidence-Based Health Care, Shuang Ho Hospital, Taipei Medical University, Taipei, Taiwan

**Keywords:** 3D body scanner, Surface topography, Scoliosis, Cobb angle

## Abstract

**Background:**

Adult scoliosis, which is characterized by a persistent lateral deviation of the spine of at least 10° in the frontal plane along with vertebral rotation in adulthood, can result from various causes, including degenerative changes, untreated childhood scoliosis, spinal trauma, and prior surgeries. Traditionally, spinal curvature is assessed by measuring the Cobb angle via radiographic imaging; however, concerns over radiation exposure have prompted exploration of alternative diagnostic tools.

This study aims to evaluate the effectiveness of a newly developed three-dimensional (3D) body scanner, equipped with 12 depth cameras, in assessing spinal alignment and measuring the Cobb angle in patients with adult scoliosis, in comparison with radiographic imaging.

**Methods:**

In this prospective cohort study, 40 patients with adult scoliosis—both idiopathic and degenerative—underwent evaluation using both radiographic imaging and 3D body scanning. Cobb angles were measured by both methods. Pearson and Spearman’s rank correlation coefficients were calculated to assess the linear and monotonic relationships between measurements. Measurement accuracy was quantified using the mean bias from Bland–Altman analysis and spatial agreement of spinal positions was further evaluated using the Intersection over Union (IoU) metric. Univariable and multivariable regression analyses were performed to assess whether body habitus (body mass index, waist circumference, waist-to-height ratio, age, and sex) influenced the absolute error between 3D body scanner-predicted and radiographic Cobb angles.

**Results:**

Cobb angle measurements obtained from 3D body scanning were highly correlated with those from radiography (Pearson *r* = 0.92, *P* < 0.001; Spearman *ρ* = 0.85, *P* < 0.001), indicating strong linear and monotonic agreement. Bland–Altman analysis showed a small mean bias of −1.06 (95% limits of agreement: −10.25 to 8.12). The average IoU was 0.89, indicating substantial spatial agreement in spinal position predictions. Importantly, obesity indices (body mass index, waist circumference, waist-to-height ratio) were not significantly associated with the absolute error between 3D body scanner-predicted and radiographic Cobb angles in either univariable or multivariable analyses.

**Conclusions:**

The 3D body scanner exhibits promise for assessing spinal alignment and measuring the Cobb angle in patients with adult scoliosis, offering a reliable alternative to traditional radiographic methods. Its accuracy was not affected by obesity-related indices, supporting its applicability across diverse patient body types. Future research should focus on refining scanning protocols and integrating patient-reported outcomes to enhance clinical utility.

## Introduction

Adult scoliosis (Aebi, 2005) is a condition characterized by a lateral deviation of the spine of at least 10° in the frontal plane along with vertebral rotation that develops or persists into adulthood. It affects a diverse population and has various etiologies and clinical presentations. Unlike adolescent idiopathic scoliosis (Weinstein et al., 2008), which typically arises during growth spurts, adult scoliosis can result from degenerative changes in the spine, previous untreated childhood scoliosis, or spinal trauma or surgery.

Scoliosis has traditionally been assessed using radiographic imaging, particularly through measurement of the Cobb angle—a crucial parameter used to quantify the degree of spinal curvature. The Cobb angle (Jin et al., 2022) is determined by measuring the angle between the extended lines of the upper end plate of the most tilted vertebral body within a curved segment and the lower end plate of the most tilted vertebral body immediately below it. However, concerns regarding cumulative radiation exposure from repeated imaging procedures have prompted researchers to explore alternative diagnostic modalities.

In recent years, three-dimentional (3D) body scanner technology (Fortin et al., 2010; Grant et al., 2019; Knott et al., 2016; Yıldırım et al., 2021) has emerged as a promising noninvasive approach for reconstructing the 3D structure of the spine and evaluating spinal deformities in patients with adult scoliosis. One such 3D body scanner developed by What’s Up Technology, Inc. utilizes multiple depth cameras to capture detailed 3D images of the torso, providing comprehensive views of spinal curvature and deformities without the use of ionizing radiation. The 3D body scanner generates precise anatomical measurements and visualizations and offers key advantages in terms of patient safety, comfort, and imaging accessibility.

Compared with radiography or EOS imaging (Wybier & Bossard, 2013), which provide two- or three-dimensional reconstructions of internal bony structures, and with magnetic resonance imaging, which captures detailed internal anatomy but is costly, the 3D body scanner is a surface-based tool that estimates spinal alignment indirectly from external body contours. In contrast to ultrasound, which can only capture localized areas, the 3D body scanner acquires comprehensive, large-area data of the entire torso in a single scan. Although it does not visualize internal structures as these modalities do, it offers several unique advantages. The scanner enables rapid, non-ionizing, and repeatable assessment of spinal alignment and curvature, making it particularly suitable for frequent monitoring and follow-up of patients with scoliosis. In this way, it serves as a complementary tool to conventional imaging, providing safe, noninvasive, and large-scale information on spinal posture and curvature.

The current study compared the effectiveness of radiographic imaging and the aforementioned 3D body scanner in assessing scoliosis among adult patients. The evaluation focused on the accuracy and reliability of both modalities in measuring the Cobb angle and characterizing spinal deformities across different segments of the spine.

## Materials and Methods

### Study design

This prospective study was conducted at Shuang Ho Hospital between December 12th 2022 and May 11th 2023. It was approved by the Institutional Review Board of Taipei Medical University (IRB no. N202207071), and informed written consent was obtained from all participants.

### Participants

A total of 40 participants were recruited from Shuang Ho Hospital. Individuals were included in the study if they were older than 20 years and had received a diagnosis of adult scoliosis, exhibiting frontal deformity with a Cobb angle greater than 10°. Individuals were excluded from the study if they had undergone spine surgery, were unable to undergo both radiography and 3D body scanning, or had failed to complete an examination within the specified timeframe. Radiographs and 3D body scans were conducted within 3 months of each other.

### 3D body scanner setup and data acquisition

The 3D body scanner used in this study was developed by What’s Up Technology, Inc. It was custom-built with 12 synchronized depth cameras arranged circumferentially around the subject to capture the front, back, and sides simultaneously. Participants were instructed to stand at the center of the scanner in an upright posture with their feet on designated positioning markers. Their arm and shoulder positions were stabilized using handles on the scanner. Each scan was completed within 5 s, producing depth images that were fused into a dense, high-resolution point cloud representing the entire body surface.

**Figure 1:**
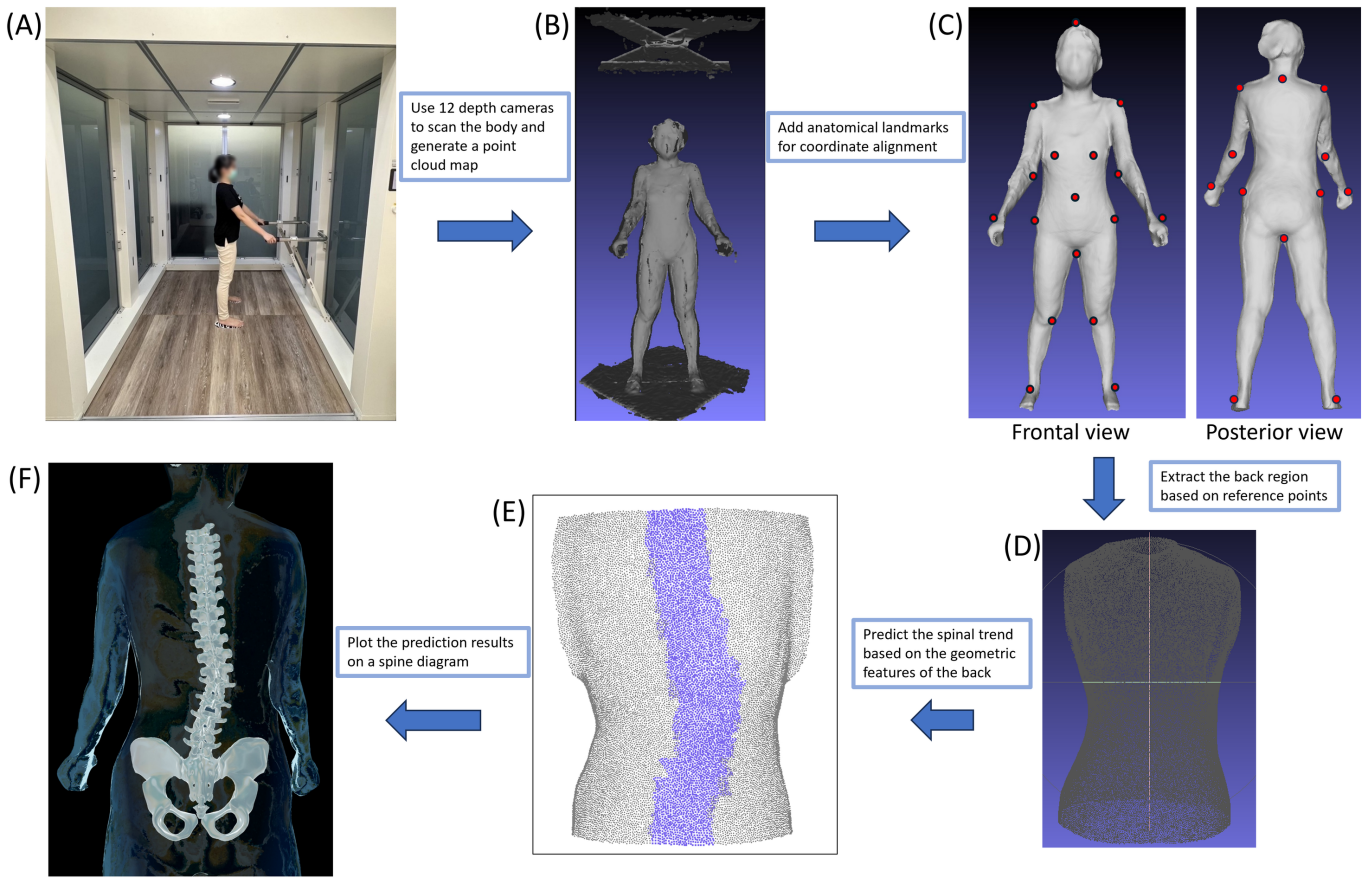
3D body scanner and 3D reconstruction process. (A) The subject stands in an upright position inside the 3D scanning scanner with arms extended forward to allow unobstructed torso capture. (B) Raw 3D point cloud data of the whole body generated by the 3D scanning scanner. (C) Reconstruction of the 3D body model with anatomical landmarks (red dots) automatically identified on both anterior and posterior surfaces. (D) Isolation of the posterior torso region according to the reference points. (E) Predicted spinal alignment derived from the posterior torso surface. (F) Three-dimensional reconstruction of the spine derived from surface geometry.

From this point cloud, anatomical landmarks—including the seventh cervical vertebra, acromial angles of the shoulders, iliac crests, and other reference points—were automatically detected by a company-developed algorithm. These landmarks were used to segment the posterior torso surface and to align the predicted spinal curve with radiographic data (Aroeira et al., 2016; Patias et al., 2010; Roy, Grünwald & Lampe, 2022; Roy et al., 2019). The spinal midline trajectory was then estimated by analyzing geometric features of the back surface, including local curvature, symmetry, and the distribution of dorsal surface points. A spline curve was fitted along this midline to generate a smooth surface-derived spinal curve, which was projected onto the coronal plane for Cobb angle calculation. Figure 1 illustrates the detailed reconstruction process of the 3D body scanner.

### Cobb angle analysis

The Cobb angle, a critical indicator in the assessment of scoliosis, was independently calculated for each participant from their 3D body scanner images and X-ray radiographs. Radiographic Cobb angles were measured by an experienced physiatrist with expertise in spinal imaging. To compute the Cobb angle from the reconstructed spinal curve, the curve was first discretized into a grid-based representation. Tangent lines were fitted along the superior and inferior borders of the curve, and the angle formed at the intersection of these tangents was defined as the Cobb angle. The primary outcome of this study was a comparison of the Cobb angle measured radiographically with that measured from the 3D body scanner images.

### Intersection over union and euclidean distance metrics

**Figure 2:**
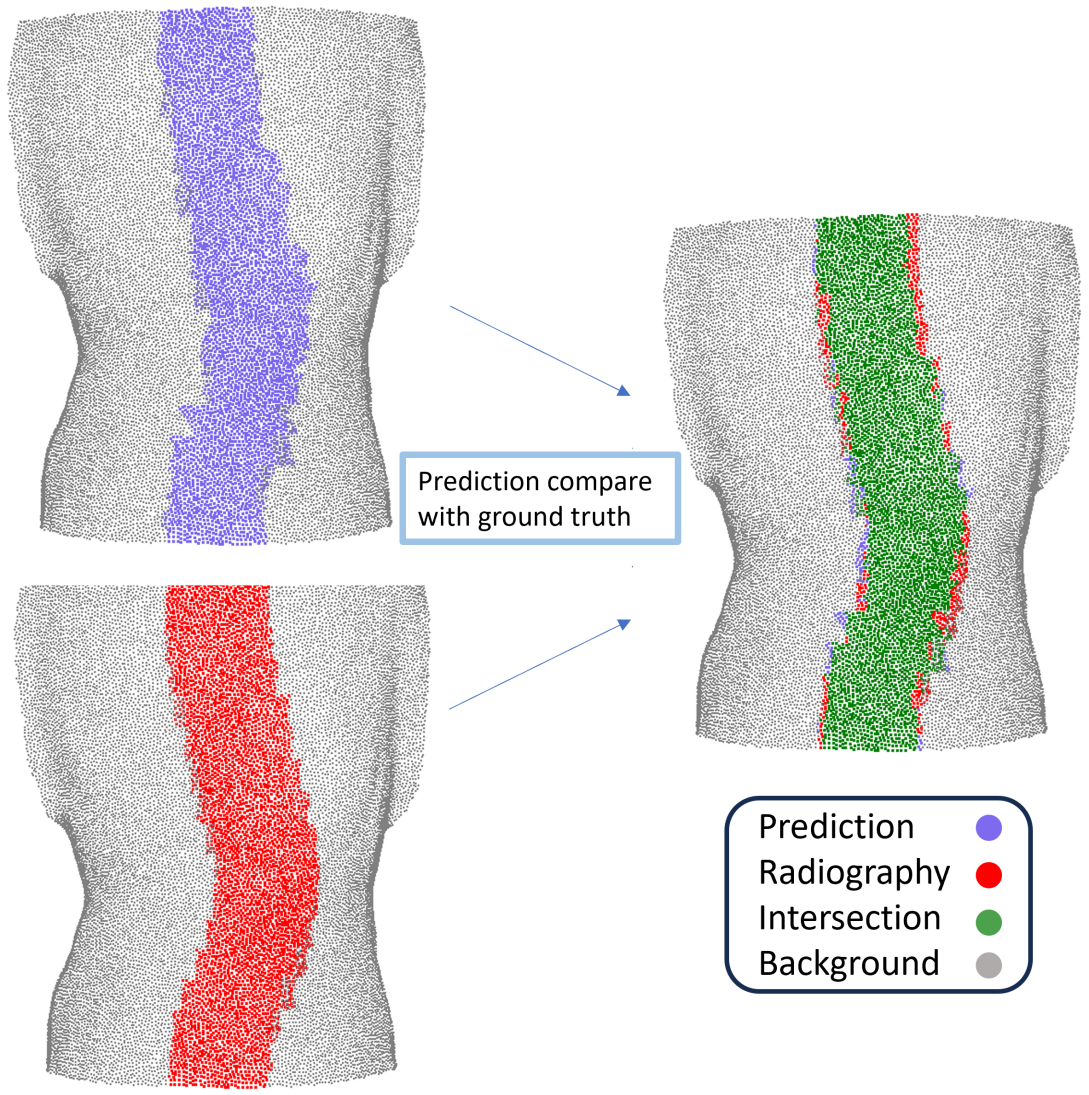
Illustration of Intersection over Union (IoU) between the predicted spinal curve from the 3D body scanner and the radiographic reference curve.

The accuracy of predicting spinal positions was evaluated using the Intersection over Union (IoU) and Euclidean distance metrics. IoU is a commonly used metric in computer vision tasks for assessing the spatial overlap between the predicted and ground truth regions. The surface-based spinal curve was reconstructed from geometric features of the posterior torso point cloud by analyzing local curvature, symmetry, and the spatial distribution of dorsal surface points, followed by spline fitting of the estimated spinal midline. For the radiographic reference, vertebral centroids were manually annotated on X-ray images by an experienced physiatrist and interpolated into a spline curve. To ensure both curves were expressed in the same spatial frame, they were aligned using anatomical landmarks including the seventh cervical vertebra, acromial angles of shoulders, natal cleft, and so on Patias et al. (2010). To enable region-based comparison, the radiographic curve was dilated with a fixed width to form a reference region, and the surface-based predicted curve was mapped to the midline area of the back. Their overlap corresponds to the green region in Fig. 2, and the IoU was computed based on the intersection and union.

The IoU is calculated as follows: IoU = Area of overlap (green region)/Area of union of the predicted spinal area (purple region) and the radiographic reference area (red region) (Fig. 2).

“Area of overlap” denotes the intersection area between the predicted and actual spinal positions, whereas “Area of union” represents the combined area encompassing both the predicted and actual positions. A perfect IoU score of 1 implies complete overlap between the prediction and the ground truth, indicating that the performance of the algorithm is excellent. Conversely, an IoU score of 0 signifies no overlap, indicating poor algorithm performance (Yu et al., 2022).

The geometric discrepancy between corresponding points in the predicted and actual spinal positions was quantified using Euclidean distance, which is the spatial difference in position between two points in a multidimensional space and provided insights into the accuracy and precision of the predicted positions.

### Statistically analysis

The characteristics of the participants are presented using descriptive statistics, including means and standard deviations. The degree of association between Cobb angles estimated using radiography and 3D body scanner measurements was assessed using both Pearson’s correlation coefficient (PCC) and Spearman’s rank correlation. PCC was used to evaluate the linear relationship between the two continuous variables, while Spearman’s rank correlation was applied to account for potential non-normality and outliers, providing a nonparametric measure of monotonic association. Correlation coefficients were interpreted as follows: <0.25 indicated little or no relationship, 0.25 to 0.50 indicated a fair relationship, 0.50 to 0.75 indicated a moderate-to-good relationship, and >0.75 indicated a good-to-excellent relationship (Portney, 2020). *P*-value < 0.05 indicated statistical significance.

In addition, a Bland–Altman analysis was performed to assess the agreement between the two measurement methods by estimating the mean bias and the 95% limits of agreement. This analysis allowed evaluation of whether the two methods could be used interchangeably.

To evaluate whether obesity and body habitus influenced the 3D body scanner measurements, univariable linear regression were first performed using the absolute error between 3D body scanner-predicted and radiographic Cobb angles as the dependent variable, and anthropometric variables, including body mass index (BMI), weight, height, waist circumference, waist-to-height ratio, chest circumference, shoulder width, age, and sex (dummy-coded as male = 1, female = 0) as predictors.

To specifically evaluate the impact of obesity on scanner accuracy, multivariable linear regression analyses were conducted using BMI as primary predictors, adjusting for age and sex. Weight and height were excluded from the primary models due to their strong collinearity with BMI and their role as indicators of body size rather than adiposity. Sensitivity analyses were performed by replacing BMI with waist circumference or waist-to-height ratio to test whether the observed associations were robust across different anthropometric specifications. Variance inflation factors (VIF) were examined to assess collinearity, with values <2 indicating no multicollinearity.

All statistical analyses were performed using IBM SPSS Statistics (version 19; IBM Corp., Armonk, NY, USA).

## Result

A total of 40 participants (25 women and 15 men) were included in this study. The mean age, height, weight, and BMI of the participants were 49.45 ± 18.06 years, 162.36 ± 8.36 cm, 62.26 ± 11.52 kg, and 23.57 ± 3.76 kg/m^2^, respectively. Among the participants, 28 had C-shaped scoliosis with a mean Cobb angle of 19.98° ± 11.45°, whereas 12 had S-shaped scoliosis with a mean major curve angle of 17.88° ± 5.82° (Table 1).

**Figure 3:**
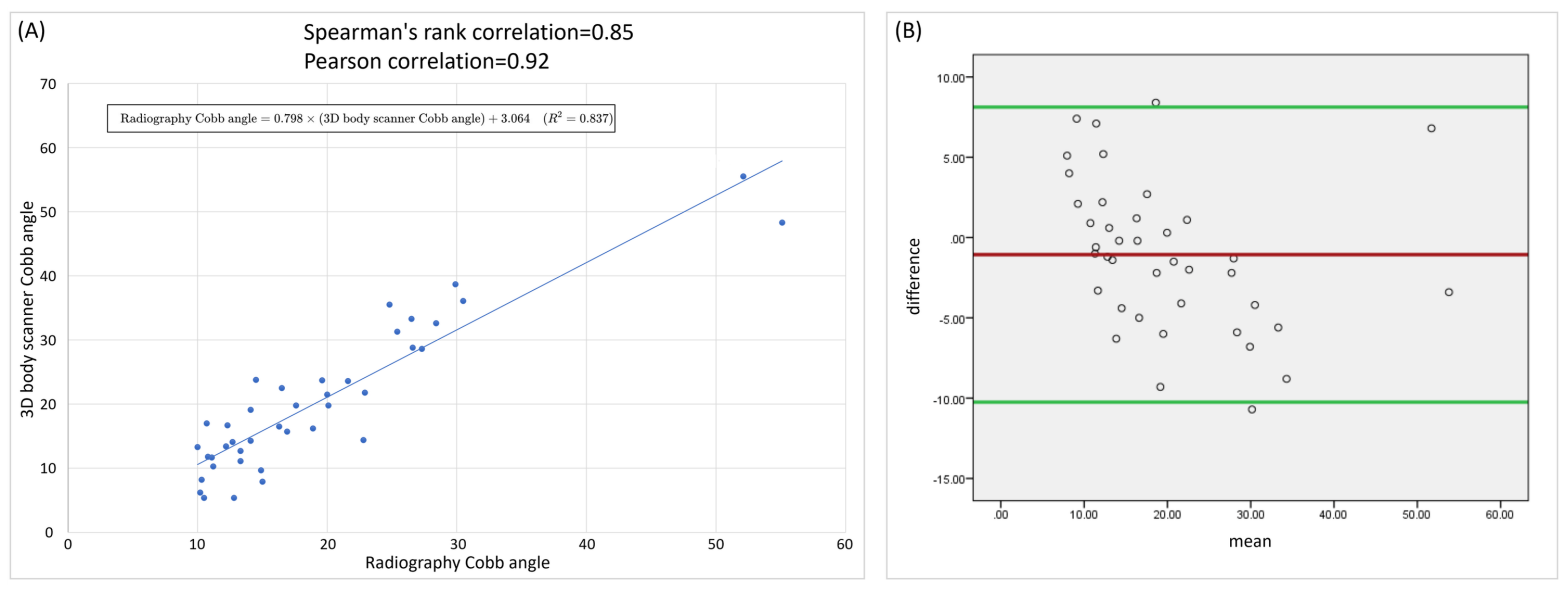
(A) Comparison of Cobb angles measured by radiographs and the 3D body scanner. (B) Bland–Altman analysis of agreement between Cobb angles measured using the 3D body scanner and radiography.

The Cobb angles measured using the 3D body scanner were highly correlated with those measured using the radiography, with a PCC of 0.915 (*P* < 0.001; Fig. 3A), indicating strong linear agreement between the two measurement methods. The PCC was 0.930 (*P* < 0.001) for C-shaped scoliosis and 0.902 (*P* < 0.001) for S-shaped scoliosis. A linear regression analysis yielded the equation: Radiography Cobb angle = 0.798 × (3D body scanner Cobb angle) + 3.064 (*R*^2^ = 0.837), showing that scanner-derived values closely tracked radiographic measurements, with radiography values being on average slightly lower.

Spearman’s rank correlation coefficient confirmed these findings, showing a strong positive monotonic relationship between the two measurement methods, with *ρ* = 0.847 (*P* < 0.001; Fig. 3A) overall. The Spearman’s rank correlation coefficients were (*P* < 0.001) 0.846 for C-shaped scoliosis and 0.782 (*P* = 0.003) for S-shaped scoliosis, indicating that the agreement remained strong even when accounting for potential non-normality and outliers.

A Bland–Altman analysis was conducted to further evaluate agreement between the two measurement methods. The mean bias was −1.06, indicating a slight tendency for radiography measurements to be lower than those from the 3D body scanner. The 95% limits of agreement ranged from −10.25 to 8.12 (Fig. 3B). Most measurements fell within these limits, with only two values exceeding the bounds, indicating occasional individual discrepancies rather than systematic differences. Taken together, these results suggest that radiography and the 3D body scanner produce highly comparable Cobb angle measurements, with the relationship between the two methods being largely linear and consistent.

**Table 1:** Characteristics of participants.

Total participants	40
Age, years	49.45 ± 18.06
Sex	
Male	15
Female	25
Height, cm	162.36 ± 8.36
Weight, kg	62.26 ± 11.52
Body mass index, kg/m^**2**^	23.57 ± 3.76
Chest circumference, cm	90.93 ± 32.10
Waist circumference, cm	85.88 ± 30.02
Cobb angle	
X-ray measured Cobb’s angle	19.35° ± 10.07°
3D body scanner estimated Cobb’s angle	20.41° ± 11.54°

**Notes.**

Categorial variables are reported as numbers, and continuous variables are reported as means ± standard deviations.

**Figure 4:**
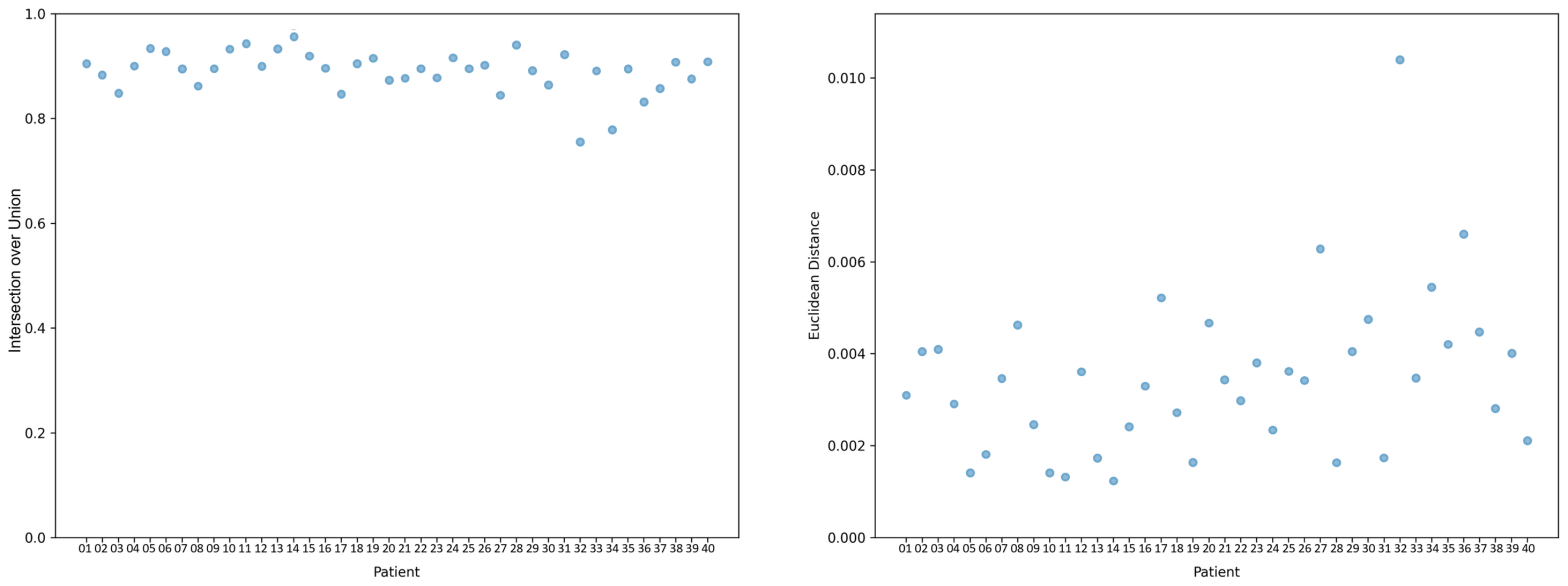
IoU and Euclidean distance (m) for each participant.

**Figure 5:**
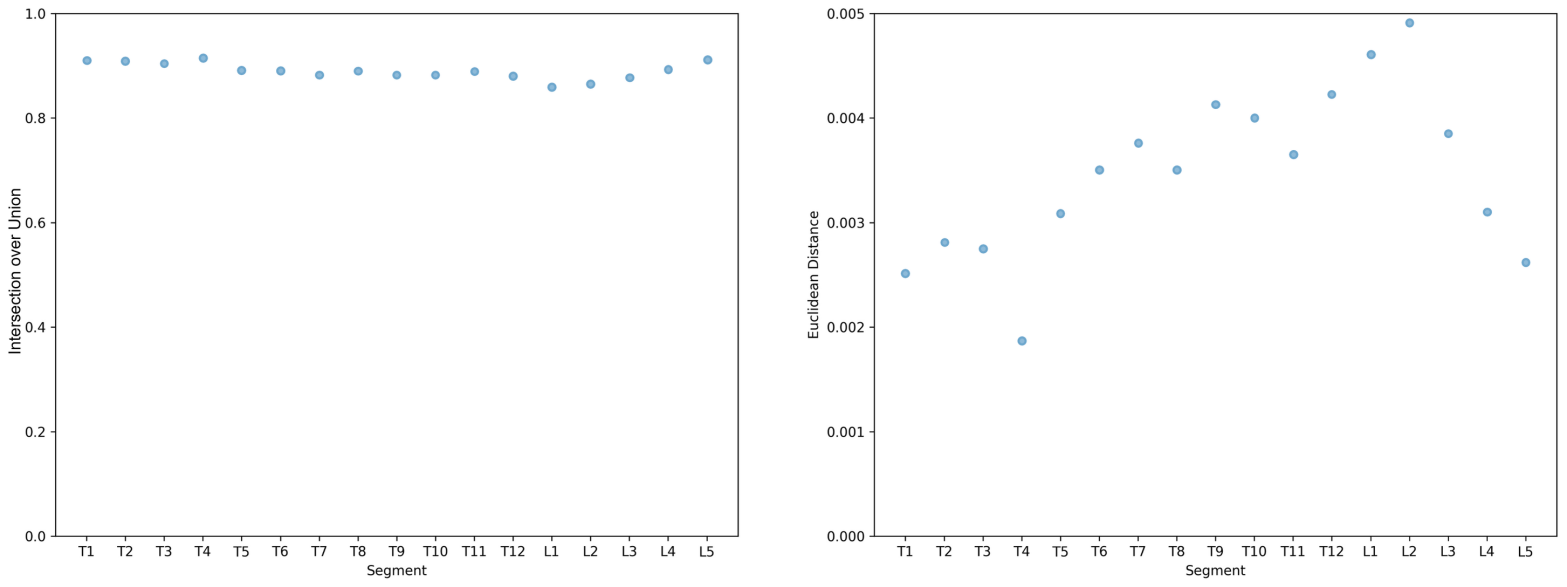
Average IoU and Euclidean distance of each vertebral segment.

The alignment and statistical measures between radiographic imaging and the 3D body scanner were compared. The average IoU and Euclidean distance for all 40 participants were 0.89 and 0.346 cm, respectively (Fig. 4). Segmented results from vertebrae T1 to T12 and L1 to L5 indicate that the ranges of average IoU and average Euclidean distance for each vertebral segment were 0.056 and 0.00304 m, respectively (Fig. 5).

In univariable analyses, BMI (*β* = −0.051, *p* = 0803), body weight (*p* = 0.184), waist circumference (*p* = 0.729), waist-to-height ratio (*p* = 0.256), chest circumference (*p* = 0.802), shoulder width (*p* = 0.342), age (*p* = 0.819), and sex (*p* = 0.474) were not significantly associated with absolute error between scanner-predicted and radiographic Cobb angles (Table 2).

**Table 2:** Univariable regression analyses of anthropometric factors on the absolute error between 3D body scanner-predicted and radiographic Cobb angles.

Predictor	*β* (Unstandardized)	Std. error	Standardized *β*	*p*-value	R^2^ (model)
Body mass index	−0.051	0.202	−0.041	0.803	0.002
Weight	−0.087	0.064	−0.214	0.184	0.046
Waist circumference	0.024	0.068	0.056	0.729	0.003
Waist-to-height ratio	11.981	10.398	0.184	0.256	0.034
Chest circumference	0.023	0.092	0.041	0.802	0.002
Shoulder width	−0.234	0.243	−0.154	0.342	0.024
Age	0.010	0.042	0.037	0.819	0.001
Sex	1.113	1.540	0.116	0.474	0.014

In the primary multivariable model, BMI was not significantly associated with absolute error between 3D body scanner-predicted and radiographic Cobb angles (*β* = −0.020, *p* = 0.933) after adjusting for age and sex. Sensitivity analyses replacing BMI with waist circumference or waist-to-height ratio yielded consistent findings, with waist circumference (*β* = 0.074, *p* = 0.424) and waist-to-height ratio (*β* = 17.785, *p* = 0.170) remaining non-significant. VIF for all models were <2, indicating no evidence of multicollinearity (Table 3).

**Table 3:** Multivariable linear regression of obesity indices adjusted for age and sex.

Predictor	*β* (Unstandardized)	Standard error	95% Confidence interval for *β*	*p*-value
Body mass index	–0.020	0.240	−0.507 to 0.466	0.933
Waist circumference	0.074	0.091	−0.112 to 0.259	0.424
Waist-to-height ratio	17.785	12.686	−7.944 to 43.513	0.170

## Discussion

This study demonstrated the efficacy of the newly developed 3D body scanner in accurately predicting spinal positions and measuring the Cobb angle in patients with scoliosis. Cobb angle measurements obtained from the 3D body scanner were highly comparable to those derived from radiographic imaging, with both Pearson and Spearman correlation analyses confirming strong linear and monotonic relationships between the two methods. Bland–Altman analysis further indicated only a small mean bias, with most values falling within the 95% limits of agreement, suggesting that the two approaches are largely interchangeable for Cobb angle estimation. This strong correlation suggests that the 3D body scanner can serve as a viable alternative or adjunct to conventional imaging techniques, potentially reducing the need for radiation exposure and providing an option that enables more frequent monitoring for patients with scoliosis.

Importantly, while previous studies (Roy, Grünwald & Lampe, 2022; Roy et al., 2019) have used 3D body scanners to assess spinal posture or curvature, few have directly compared scanner-predicted Cobb angles with radiographic measurements. By performing this quantitative comparison, our study provides novel evidence on the accuracy and potential clinical utility of multi-camera depth-based 3D scanning for adult scoliosis assessment, supporting its use as a noninvasive, radiation-free monitoring tool.

The high average IoU across all participants indicates substantial spatial agreement between the predicted and actual spinal positions. This metric is critical in clinical settings, where precise anatomical alignment is essential for accurate diagnosis and treatment planning. The finding of a low average Euclidean distance of 0.346 cm further supports the scanner’s ability to minimize geometric discrepancies.

However, relatively low IoU and high Euclidean distances were noted for participants 32 and 34. For participant 34, who had degenerative scoliosis, these discrepancies likely occurred because of the complex nature of degenerative changes affecting spinal morphology. Participant 32 demonstrated a spinal curve that was consistent with the radiographic findings but had lower IoU and higher Euclidean distance values, indicating potential problems with respect to accurately capturing the exact spatial position of the spine. This observation aligns with the findings of Frerich et al. (2012), who stated that surface topographical assessment, despite providing valuable insights into spinal curvature and asymmetry, cannot replace radiographic analysis when patients with adolescent idiopathic scoliosis are being monitored because of its inability to directly evaluate bone morphology.

Segmented analysis focusing on vertebrae T1 to T12 and L1 to L5 revealed varying IoU and Euclidean distance values, reflecting challenges related to accurately predicting spinal curvature across different segments. Our study obtained lower IoU values, particularly in segments L1 and L2, which may be attributable to the specific characteristics of the participants included, with some exhibiting present curvature near these vertebrae. Previous studies have indicated that 3D noncontact surface scanning may not be accurate in mapping clinically relevant coronal plane spinal contours (Goldberg et al., 2001; Kotwicki et al., 2007). Our findings are consistent with these concerns, suggesting that although the 3D body scanner offers substantial benefits in terms of noninvasiveness and comprehensiveness of anatomical visualization, its application may be more challenging in regions where the spinal curvature is pronounced or complex.

Compared with traditional optical surface scanners and laser profilometry systems (Krawczyk & Sitnik, 2023; Markiewicz et al., 2017; Miles, 2018), our multi-camera depth-based system provides several advantages. The use of 12 synchronized depth cameras enables comprehensive posterior coverage with minimal blind spots, ensuring reliable acquisition even in participants with asymmetric posture or larger body habitus. Because depth sensors directly capture 3D coordinates rather than relying on surface texture or illumination, the resulting point clouds maintain high geometric fidelity and reduce reconstruction errors caused by lighting conditions or clothing.

Furthermore, the integration of multiple views facilitates robust detection of anatomical landmarks such as the seventh spinous process and iliac crest, which are essential for accurate alignment of the surface-derived and radiographic curves. Importantly, our computational pipeline differs from prior approaches that often relied on simple surface symmetry or two-dimensional projections. By extracting the spinal midline from the dorsal surface point cloud and fitting it with a spline curve, our algorithm more precisely captures subtle spinal trends and provides closer correspondence with radiographic references. This methodological improvement enhances both spatial alignment and quantitative comparison. In addition, the synchronized multi-camera capture minimizes motion artifacts, shortens acquisition time, and ensures reproducibility across repeated measurements, making the system well suited for both clinical and research applications.

Ghaneei et al. (2019) reported on the problem of fatty tissue potentially obscuring the underlying bony anatomy during surface scanning assessments of spinal bone positions. This observation aligns with findings from Little et al. (2019), who noted inconsistencies between the spinous process and surface markers in the lower lumbar spine and over the gluteal muscles. The study indicated that a higher BMI and increased adipose tissue thickness may lead to challenges in accurately determining the position of internal spinal bones from visible surface anatomy. While greater adipose tissue thickness may pose challenges for surface scanning, the current study suggests that obesity, as assessed by BMI and waist-based indices, does not affect the absolute error between 3D body scanner-predicted and radiographic Cobb angles. This finding was consistent in both univariable and multivariable analyses, even after adjustment for age and sex. One possible explanation is that the system primarily analyzes posterior torso geometry and midline deviation rather than absolute tissue thickness. Fat accumulation predominantly affects the anterior trunk and extremities, while posterior spinal contours remain relatively preserved. Furthermore, the depth cameras capture surface geometry independent of tissue composition, and the algorithm relies on proportional asymmetry rather than absolute body size. Because the error remained stable across patients with different body habitus, the technology appears robust and reliable for individuals regardless of whether they are lean or overweight. This supports the applicability of the scanner across a broad range of patient body types and suggests that variations in fat distribution do not interfere with posterior torso geometry extraction or spinal curve reconstruction.

A systemic review by Su et al. (2022) highlighted surface topography techniques as a promising approach for assessing scoliosis, indicating that they can complement or even replace traditional radiological measurements such as the Cobb angle. Although several studies have demonstrated good-to-strong correlations between surface topography measurements (*e.g.*, scoliosis angles and certain asymmetry parameters) and the Cobb angle, significant discrepancies between the obtained results have been noted. These inconsistencies are particularly evident in measurements involving rotational aspects or other asymmetry parameters not directly captured by the Cobb angle, underscoring the multifaceted nature of scoliosis assessment, where the internal skeletal alignment may not always mirror external aesthetic concerns (James, 1954).

Despite offering advantages in terms of assessing external shape and asymmetries across various anatomical landmarks, no gold standard for surface topography parameters has been established, whereas the Cobb angle has been established in radiology. Our study employed consistent methodologies and a relatively large sample size to establish the reliability and validity of surface topography parameters in clinical practice.

In addition to playing a diagnostic role in scoliosis, the Cobb angle serves as a crucial metric for evaluating the outcomes of both surgical and conservative treatments. Although clinicians typically prioritize internal alignment measured through the Cobb angle, patients and their families often place greater emphasis on improving the external appearance and aesthetics of the spine. The International Society on Scoliosis Orthopaedic and Rehabilitation Treatment (Negrini et al., 2006) advocates for a patient-centered approach to scoliosis, emphasizing that treatment strategies should address current symptoms and future needs, including aesthetic concerns. The importance of 3D body scanner technologies becomes evident when one considers the positive influence of enhancing the outward appearance of the spine on a patient’s quality of life. Therefore, in addition to traditional radiological metrics, future research should consider patient-reported outcomes regarding appearance and quality of life to provide a more comprehensive assessment of 3D body scanners. Additionally, future studies could employ longitudinal research designs to assess the performance of such scanners over time and evaluate their effectiveness in monitoring disease progression and treatment outcomes.

The main limitation of our study is its inclusion of participants with idiopathic scoliosis as well as those with *de novo* or degenerative scoliosis, which led to the scoliosis of our study having numerous causes. Additionally, the ages of the patients varied significantly. Most participants had relatively mild-to-moderate scoliosis, with Cobb angles below 30°, which limits the generalizability of the results to patients with more severe curves. Moreover, the timing of radiographic imaging and 3D body scanner sessions varied, potentially introducing bias because the Cobb angle may fluctuate over time. However, this bias may have been minimized by the fact that all participants were adults, in whom spinal curvature changes are typically less pronounced relative to younger individuals.

## Conclusion

Our study highlights the efficacy of 3D body scanner technology in accurately predicting spinal positions and measuring the Cobb angle in patients with adult scoliosis. The high correlation between the measurements derived from the 3D body scanner and those derived through radiographic imaging highlights the reliability of the scanner’s measurements, suggesting it as a viable alternative or complement to traditional X-ray methods. Use of such scanners not only reduces the need for radiation exposure but also allows for an increase in the monitoring frequency for patients with scoliosis. Importantly, our analyses demonstrated that the absolute error between 3D body scanner-predicted and radiographic Cobb angles was not affected by obesity-related indices such as BMI, waist circumference, or waist-to-height ratio, supporting its applicability across patients with different body types.

While these results are promising, the performance of the scanner in cases of more severe scoliosis remains to be validated. Nonetheless, this radiation-free technology holds potential value for scoliosis screening and early detection, particularly in patients whose curves fall within the mild-to-moderate range observed in this study. Challenges remain in cases of degenerative scoliosis and specific spinal segments, highlighting the need for further research. Future research should focus on refining protocols and integrating patient-reported outcomes to further validate the clinical utility and patient-centered benefits of 3D scanner technology. Additionally, longitudinal studies assessing the scanner’s performance over time could enhance its applicability for monitoring disease progression and treatment outcomes.

##  Supplemental Information

10.7717/peerj.20752/supp-1 Supplemental Information 1 Raw data

10.7717/peerj.20752/supp-2 Supplemental Information 2 Codebook

10.7717/peerj.20752/supp-3 Supplemental Information 3 STROBE

## Additional Information and Declarations

### Data Availability

The following information was supplied regarding data availability: The raw data is available in the Supplemental File.
